# A randomized, 3-arm, neoadjuvant, phase 2 study comparing docetaxel + carboplatin + trastuzumab + pertuzumab (TCbHP), TCbHP followed by trastuzumab emtansine and pertuzumab (T-DM1+P), and T-DM1+P in HER2-positive primary breast cancer

**DOI:** 10.1007/s10549-020-05524-6

**Published:** 2020-01-17

**Authors:** Norikazu Masuda, Shoichiro Ohtani, Toshimi Takano, Kenichi Inoue, Eiji Suzuki, Rikiya Nakamura, Hiroko Bando, Yoshinori Ito, Kazushige Ishida, Takashi Yamanaka, Katsumasa Kuroi, Hiroyuki Yasojima, Hiroi Kasai, Tsuyoshi Takasuka, Takaki Sakurai, Tatsuki R. Kataoka, Satoshi Morita, Shinji Ohno, Masakazu Toi

**Affiliations:** 1 Department of Surgery, Breast Oncology, National Hospital Organization Osaka National Hospital, Osaka, Japan; 2 Department of Breast Surgery, Hiroshima City Hiroshima Citizens Hospital, Hiroshima, Japan; 3 Department of Medical Oncology, Toranomon Hospital, Tokyo, Japan; 4 Division of Breast Oncology, Saitama Cancer Center, Saitama, Japan; 5 Department of Breast Surgery, Kyoto University Hospital, Kyoto, Japan; 6 Division of Breast Surgery, Chiba Cancer Center, Chiba, Japan; 7 Breast and Endocrine Surgery, Faculty of Medicine, University of Tsukuba, Tsukuba, Japan; 8 Breast Medical Oncology Department, Cancer Institute Hospital of Japanese Foundation for Cancer Research, Tokyo, Japan; 9 Department of Surgery, Iwate Medical University, Morioka, Japan; 10 Department of Breast and Endocrine Surgery, Kanagawa Cancer Center, Yokohama, Japan; 11 Department of Breast Surgery, Tokyo Metropolitan Health and Hospitals Corporation Ebara Hospital, Tokyo, Japan; 12 Institute for Advancement of Clinical and Translational Science, Kyoto University Hospital, Kyoto, Japan; 13 Oncology Lifecycle Management Department, Chugai Pharmaceutical Co., Ltd., Tokyo, Japan; 14 Department of Diagnostic Pathology, Kyoto University Hospital, Kyoto, Japan; 15 Department of Biomedical Statistics and Bioinformatics, Kyoto University Graduate School of Medicine, Kyoto, Japan; 16 Breast Oncology Center, Cancer Institute Hospital of Japanese Foundation for Cancer Research, Tokyo, Japan; 17 Breast Cancer Unit, Kyoto University Hospital, Kyoto University, 54 Shogoin-Kawaharacho, Sakyo-ku, Kyoto, 606-8507 Japan

**Keywords:** Dual HER2-targeted therapy, Neoadjuvant therapy, Pertuzumab, Trastuzumab emtansine, Pathological complete response, Safety

## Abstract

**Purpose:**

The standard of care in the neoadjuvant setting for human epidermal growth factor receptor 2 (HER2)-positive breast cancer is dual HER2-targeted therapy. However, a need to minimize treatment-related toxicity and improve pathological complete response (pCR) rates, particularly in luminal HER2-positive disease, exists.

**Methods:**

Neopeaks, a randomized, phase 2 study, compared docetaxel + carboplatin + trastuzumab + pertuzumab (TCbHP; 6 cycles; group A), TCbHP (4 cycles) followed by trastuzumab emtansine + pertuzumab (T-DM1+P; 4 cycles; group B), and T-DM1+P (4 cycles; group C) regimens in HER2‐positive primary breast cancer patients; concurrent hormone therapy with T-DM1+P was administered in case of estrogen receptor positivity (ER+). Based on tumor shrinkage, nonresponders in group C were switched to 5-fluorouracil + epirubicin + cyclophosphamide (FEC; 4 cycles). Primary endpoint was pCR (comprehensive pCR ypN0 [ypT0-TisypN0]).

**Results:**

Of 236 patients enrolled, 204 were randomized to groups A (*n* = 51), B (*n* = 52), and C (*n* = 101). In group C, 80 (79%) patients continued T-DM1+P following favorable response, whereas 21 (21%) nonresponders switched to FEC. pCR rate was numerically higher with the TCbHP →  T-DM1+P regimen (71%) versus the standard TCbHP (57%) and T-DM1+P (57%) regimens. The rate in group C was higher among responders continuing T-DM1+P (63%) versus nonresponders who switched to FEC (38%). pCR rates after initial 4 cycles of T-DM1+P (group C; 57%) and standard TCbHP regimen (57%) were equivalent. pCR rate in patients with ER+ was significantly higher in group B (69%) than groups A (43%) and C (51%), but was comparable in patients with ER− (67–76%). Compared with the T-DM1-based arm, the incidence of adverse events was higher in the taxane-based arms.

**Conclusion:**

In the neoadjuvant setting, the pCR rate with the standard TCbHP →  T-DM1+P regimen was numerically better than the TCbHP regimen alone and significantly better in patients with ER+. Personalization of the T-DM1+P regimen could serve as a reasonable approach to minimize toxicity while maintaining efficacy.

**Trial registration ID:** UMIN-CTR: UMIN000014649.

**Electronic supplementary material:**

The online version of this article (10.1007/s10549-020-05524-6) contains supplementary material, which is available to authorized users.

## Introduction

Current treatment guidelines recommend the use of multidrug chemotherapy, such as a sequential combination of an anthracycline-containing regimen and taxane or concurrent use of taxane and platinum, in combination with the anti-human epidermal growth factor receptor 2 (HER2) monoclonal antibodies trastuzumab and pertuzumab for the treatment of HER2-positive (HER2+) primary breast cancer [1, 2].

Dual HER2-targeted therapy with lapatinib or pertuzumab in combination with trastuzumab has significantly increased pathological response rates in patients with HER2+ breast cancer. For example, ypT0/is and/or ypN0 rates (pathological complete response [pCR]) showed improvement from 29.5–52.5% to 51.3–65.9% by adding lapatinib [3–6] and from 22–29% to 45.8–66.2% by adding pertuzumab [7–10]. Furthermore, attempts have been made to reduce the toxicity of chemotherapy by using dual HER2 blockade alone or trastuzumab emtansine (T-DM1) with or without pertuzumab. Dual HER2 blockade with trastuzumab and pertuzumab alone in the absence of cytotoxic chemotherapy has shown reasonable pathological response rates (16.8%) in patients with locally advanced or inflammatory, operable, HER2+ breast cancer [7]. The response rate was higher (44.4%) with the combination of T-DM1 and pertuzumab (T-DM1+P) in patients with HER2+ early breast cancer [10].

Several studies have investigated HER2 blockade with concurrent hormone therapy (HT) in patients with hormone receptor-positive (HR+) HER2+ breast cancer. A phase 3 study demonstrated reduced disease progression (hazard ratio 0.71; 95% confidence interval [CI] 0.53–0.96; *P* = 0.019) and significant clinical benefit with the lapatinib–letrozole combination in post-menopausal patients with HR +  HER2+ metastatic breast cancer [11]. A high pathological complete response (pCR, 21%; pathologic response rate, 54%) was also observed with add-on letrozole (+ luteinizing hormone-releasing hormone [LHRH] agonist in pre-menopausal women) to a 12-week trastuzumab–lapatinib combination in patients with estrogen receptor (ER) positive (ER+) HER2+ breast cancer in a phase 2 study [12]. Similarly, the trastuzumab–anastrozole combination showed prolonged progression-free survival (PFS; median, 4.8 months) in patients with HER2+ HR+ metastatic breast cancer in the phase 3 TAnDEM study [13]. In the PERTAIN study, the PFS was 18.9 months/15.8 months in patients with HER2+ HR+ metastatic breast cancer/locally advanced breast cancer who received trastuzumab + aromatase inhibitor (AI) with/without pertuzumab, respectively [14]. A systematic review concluded that treatment with lapatinib/trastuzumab + AI was clinically more effective than AI monotherapy in patients with HR +  HER2+ breast cancer [15]. Thus, concomitant HT is expected to show additional efficacy due to dual action, in ER+ HER2+ breast cancer.

Furthermore, among the common breast cancer subtypes, higher pCR rates were observed in ER-negative (ER−), luminal B HER2-negative (HER2−), nonluminal HER2+, and triple-negative (ER−, progesterone receptor [PgR]-negative, and HER2−) disease [16].

Therefore, the key considerations that remain are improving pCR rates in luminal HER2+ disease, which is less sensitive to the combination of chemotherapy and anti- HER2+ agents, and minimizing treatment-related toxicity without reducing efficacy in terms of pCR rates. This randomized, phase 2, 3-arm study was designed to compare docetaxel + carboplatin + trastuzumab + pertuzumab (TCbHP), TCbHP followed by T-DM1+P, and T-DM1+P for treating HER2+ primary breast cancer patients as a Japan Breast Cancer Research Group (JBCRG) association study-20, Neopeaks. In the T-DM1+P arm, we personalized the treatment after confirming tumor shrinkage and responders continued on the same treatment, whereas nonresponders were switched to a different type of anthracycline-based regimen.

## Materials and methods

### Study design

This randomized, phase 2, open-label, 3-arm study enrolled patients between August 2014 and February 2016 at 17 institutions/centers across Japan. Eligible patients were randomized in a 1:1:2 ratio into 3 treatment groups: group A received 6 cycles of TCbHP; group B received 4 cycles of TCbHP followed by 4 cycles of T-DM1+P; and group C (the response-guided regimen) received 4 cycles of T-DM1+P followed by 2 cycles of T-DM1+P among responders (subgroup C1) or switched to 4 cycles of 5-fluorouracil + epirubicin + cyclophosphamide (FEC) among nonresponders (subgroup C2) (Online Resource 1). Responders were defined as patients with ≥ 30% decrease in tumor size by magnetic resonance imaging (MRI) and with a Ki67 level of ≤ 10% or absence of cancer cells in core needle biopsy. Patients received the assigned regimens on day 1 of each 3-week cycle. Minimization method was used, and treatment allocation adjustment factors were ER status, menopausal status, T1–T2/T3, N0/N1, and institution. Breast surgery was performed within 10 weeks of neoadjuvant therapy completion. The study was conducted in accordance with the Declaration of Helsinki and Guidelines for Good Clinical Practice. The study was approved by the Institutional Review Board of each participating institution. Written informed consent was obtained from all patients.

### Patients

Key inclusion criteria were women aged ≥ 20 and ≤ 70 years with Eastern Cooperative Oncology Group performance status 0–1, histologically confirmed primary invasive breast cancer (cT1c–cT3, cN0–cN1, cM0), targeted lesion size ≤ 7 cm by MRI or ultrasound imaging, and appropriately maintained organ function. HER2 expression was determined using immunohistochemistry (IHC) and in situ hybridization (fluorescence in situ hybridization [FISH] and dual in situ hybridization [DISH] assays) by central laboratory testing at the Department of Diagnostic Pathology, Kyoto University Hospital, in accordance with the recommendations for HER2 testing in breast cancer by the American Society of Clinical Oncology/College of American Pathologists in the 2013 updated clinical practice guidelines [17]. ER expression status by IHC and Ki67 index was also investigated mandatorily at the central laboratory after obtaining written informed consent. Patients with bilateral breast cancer, axillary lymph node dissection before neoadjuvant chemotherapy, incision/excision biopsy of the primary lesion or axillary lymph node(s), multiple primary cancers, and/or grade ≥ 2 peripheral sensory neuropathy were excluded.

### Treatment

Details of dose schedule, concurrent HT, and discontinuation or treatment suspension criteria are described in Online Resource 2. Patients with ER+ were treated with concurrent HT along with T-DM1+P; pre-menopausal patients received an LHRH analog with tamoxifen, whereas post-menopausal patients received letrozole. Breast surgery (total mastectomy, partial mastectomy [Bp], or quadrantectomy [Bq]) was performed within 10 weeks of neoadjuvant therapy completion at the investigator’s discretion. Immediate breast reconstruction was allowed for mastectomy cases.

Post-operative adjuvant therapy was performed by investigator choice according to the clinical guidelines. One-year treatment was recommended for trastuzumab (including the neoadjuvant therapy or T-DM1 treatment period). When residual, invasive cancer was pathologically observed, addition of appropriate chemotherapy (e.g., anthracyclines) was recommended. In patients with ER+, HT was recommended for ≥ 5 years. Local radiation therapy was performed when deemed necessary (Online Resource 2).

### Endpoints

Primary endpoint was pCR (comprehensive pCR [CpCR]ypN0 [ypT0-TisypN0], including residual ductal carcinoma in situ) rate by central histopathological review. Exploratory analyses assessed pCR rates based on ER status. Secondary endpoints were (1) CpCR defined as noninvasive cancer or in situ tumor residuals (strict pCR [SpCR] + invasive pCR [pCRinv]); (2) SpCR (histological absence of tumor [grade 3] with pathological evidence of cancer prior to treatment + noninvasive cancer or in situ tumor residuals [pCRinv]); (3) Quasi pCR (QpCR) defined as limited number of tumor cells present in the removed breast tissue (grade 2b) [18]; and (4) QpCR + ypN0. Other endpoints included overall response rate (ORR), clinical complete response (cCR) assessed by MRI/positron emission tomography-computed tomography (PET-CT), tumor shrinkage assessed by MRI/PET-CT at cycle 4, breast conservation rate (breast-conserving surgery [BCS; Bp or Bq] with clear margin), breast conservation rate in patients who shifted from pre-planned mastectomy to BCS, disease-free survival (DFS), overall survival (OS), and safety using the Medical Dictionary for Regulatory Activities/Japanese (MedDRA/J) v18.1 and graded per the National Cancer Institute Common Terminology Criteria for Adverse Events (NCI CTCAE) v4.03.

### Statistical analysis

For groups A and B, a total of 50 patients each were set to yield 95% CIs of 45.2–73.6% and 61.8–86.9%, respectively, assuming true pCR rates of 60% and 75%, respectively. For group C, a total of 100 patients were set to yield a 95% CI of 65.3–83.1%, assuming a true pCR rate of 75%. For efficacy and safety endpoints, point estimates and 95% CIs were calculated or *χ*^2^ test was used (significance level 0.05) to compare across groups. pCR rates in ER+/− patients were compared across groups. In an exploratory analysis, pCR rates stratified by HER2 expression status (IHC3+ [strong] or IHC2+ [equivocal] plus DISH+ [mild]) were compared within the group. DFS and OS curves were estimated using the Kaplan–Meier method.

## Results

### Patient disposition and baseline characteristics

**Fig. 1:**
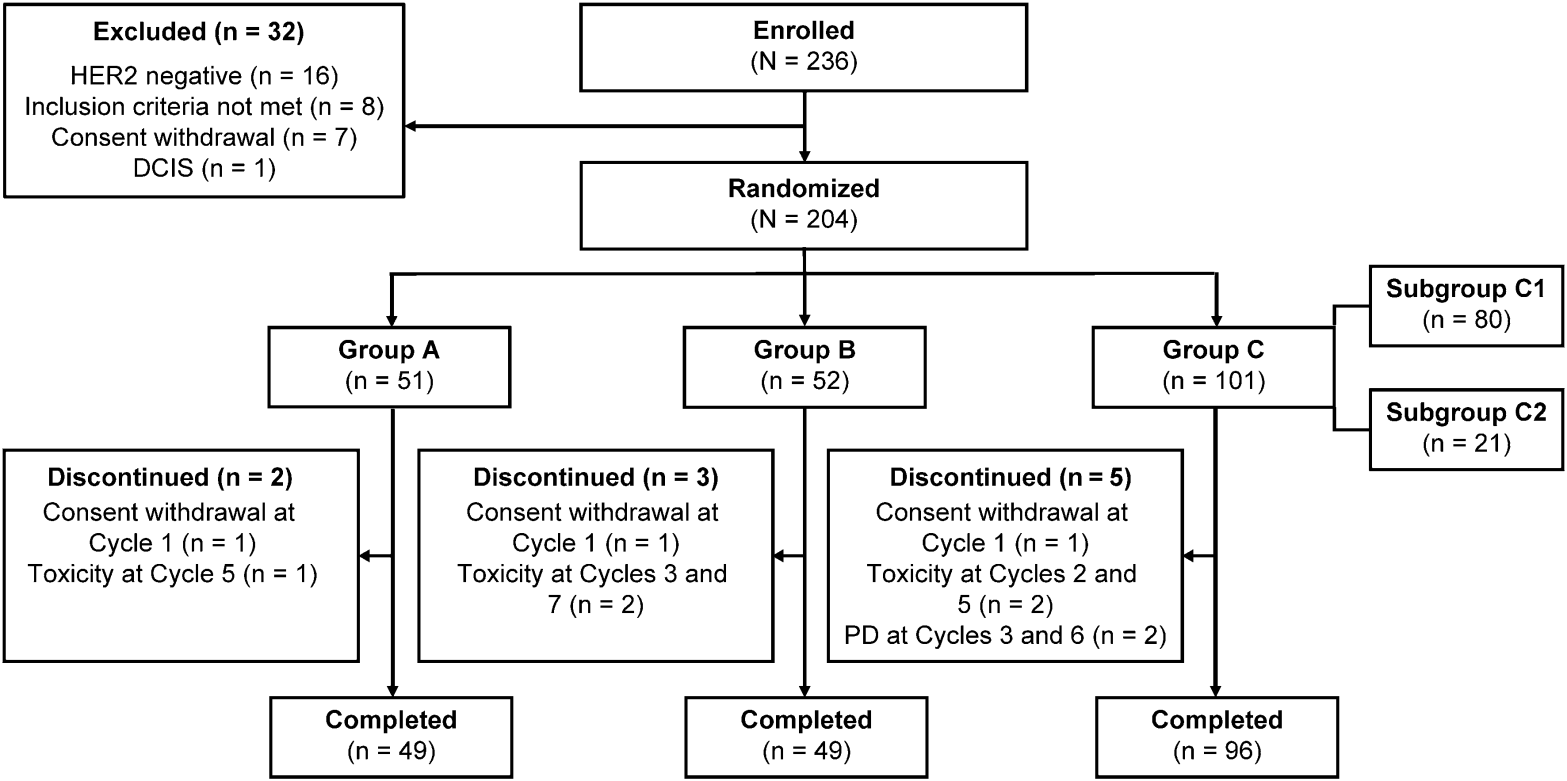
Patient disposition. *PD* progressive disease, *DCIS* ductal carcinoma in situ, *HER2* human epidermal growth factor receptor 2

Of the 236 patients primarily enrolled, 204 were randomized to groups A (*n* = 51), B (*n* = 52), and C (*n* = 101) (Fig. 1); 32 were excluded, most commonly due to HER2 negative status (*n* = 16) by central laboratory testing. Within group C, 80 (79.2%) patients continued T-DM1+P (subgroup C1) after a favorable response, whereas 21 (20.8%) nonresponders were switched to FEC (subgroup C2; Online Resource 3). Overall, 49 patients each in groups A and B and 96 patients in group C completed the protocol-specified therapy (Fig. 1). Dose reductions were required for docetaxel (relative dose intensity [RDI], 94.9%) in 10 (19.6%) patients and for carboplatin (RDI, 93.7%) in 12 (23.5%) patients in group A, for docetaxel (RDI, 95.3%) and carboplatin (RDI, 95.0%) in 9 (17.3%) patients each and T-DM1 (RDI, 97.9%) in 8 (15.4%) patients in group B, and for T-DM1 (RDI, 97.6%) in 11 (10.9%) patients in group C. Patients were generally well matched across treatment groups with a median (range) age of 53 (25–70) years and tumor size of 26 (11–70) mm. Overall, 118 (57.8%) patients were ER+ and 94 (46.1%) were post-menopausal (Table 1).

**Table 1:** Patient demographics and baseline characteristics

Variable	Overall (*N* = 204)	Group A (*n* = 51)	Group B (*n* = 52)	Group C (*n* = 101)	Subgroup C1 (*n* = 80)	Subgroup C2 (*n* = 21)
Age, years						
Median (range)	53.0 (25–70)	53.0 (28–70)	53.0 (29–69)	52.0 (25–70)	51.5 (25–70)	53.0 (40–67)
Menopausal status, *n* (%)						
Post-menopause	94 (46.1)	23 (45.1)	23 (44.2)	48 (47.5)	39 (48.8)	9 (42.9)
Pre-menopause	110 (53.9)	28 (54.9)	29 (55.8)	53 (52.5)	41 (51.3)	12 (57.1)
ECOG performance status, *n* (%)						
0	203 (99.5)	51 (100.0)	52 (100.0)	100 (99.0)	79 (98.8)	21 (100.0)
1	1 (0.5)	0 (0.0)	0 (0.0)	1 (1.0)	1 (1.3)	0 (0.0)
T stage (primary tumor), *n* (%)						
T1c	44 (21.6)	11 (21.6)	13 (25.0)	20 (19.8)	14 (17.5)	6 (28.6)
T2	144 (70.6)	37 (72.5)	35 (67.3)	72 (71.3)	58 (72.5)	14 (66.7)
T3	16 (7.8)	3 (5.9)	4 (7.7)	9 (8.9)	8 (10.0)	1 (4.8)
Tumor size by MRI/PET-CT, mm						
Median (range)	26.0 (11–70)	27.0 (11–58)	25.5 (12–56)	27.0 (11–70)	27.0 (11–70)	27.0 (12–51)
N stage, *n* (%)						
N0	129 (63.2)	34 (66.7)	31 (59.6)	64 (63.4)	49 (61.3)	15 (71.4)
N1	75 (36.8)	17 (33.3)	21 (40.4)	37 (36.6)	31 (38.8)	6 (28.6)
HER2 status, *n* (%)						
IHC3+	177 (86.8)	45 (88.2)	45 (86.5)	87 (86.1)	70 (87.5)	17 (81.0)
IHC 2+/CISH+	27 (13.2)	6 (11.8)	7 (13.5)	14 (13.9)	10 (12.5)	4 (19.0)
ER status, *n* (%)						
Positive	118 (57.8)	30 (58.8)	29 (55.8)	59 (58.4)	44 (55.0)	15 (71.4)
Negative	86 (42.2)	21 (41.2)	23 (44.2)	42 (41.6)	36 (45.0)	6 (28.6)
Ki67 index, *n* (%)						
< 10%	10 (4.9)	2 (3.9)	2 (3.8)	6 (5.9)	4 (5.0)	2 (9.5)
10% to < 20%	37 (18.1)	11 (21.6)	7 (13.5)	19 (18.8)	14 (17.5)	5 (23.8)
20% to < 30%	49 (24.0)	10 (19.6)	17 (32.7)	22 (21.8)	19 (23.8)	3 (14.3)
30% to < 50%	65 (31.9)	16 (31.4)	11 (21.2)	38 (37.6)	32 (40.0)	6 (28.6)
≥ 50%	43 (21.1)	12 (23.5)	15 (28.8)	16 (15.8)	11 (13.8)	5 (23.8)
Planned surgical procedure, *n* (%)						
Bt	128 (62.7)	33 (64.7)	31 (59.6)	64 (63.4)	50 (62.5)	14 (66.7)
Bp/Bq	76 (37.3)	18 (35.3)	21 (40.4)	37 (36.6)	30 (37.5)	7 (33.3)

*Bp* partial mastectomy, *Bq* quadrantectomy, *Bt* total mastectomy, *CISH* chromogenic in situ hybridization, *ECOG* Eastern Cooperative Oncology Group, *ER* estrogen receptor, *HER2* human epidermal growth factor receptor 2, *IHC* immunohistochemistry, *IHC2+* equivocal for HER2 protein expression (circumferential membrane staining that is incomplete, weak, or moderate within > 10% of the invasive tumor cells or complete and circumferential intense membrane staining within ≤ 10% of invasive tumor cells), *IHC3+* positive HER2 expression (circumferential membrane staining that is complete, intense, and in > 10% of invasive tumor cells), *MRI* magnetic resonance imaging, *PET-CT* positron emission tomography-computed tomography

### Post-operative adjuvant therapy

Overall, post-operative adjuvant chemotherapy was administered in 36/204 (17.6%) patients (with pCR, 5/124 [4.0%]; without pCR, 31/80 [38.8%]), with 28 (77.8%) of them receiving an anthracycline-containing regimen. By treatment groups, 10/103 (anthracyclines in 9/10) patients in groups A and B and 26/101 (anthracyclines in 19/26) in group C were administered post-operative therapy. Overall, the most common post-operative therapy administered was trastuzumab (98%, 200/204), and concomitant HT (54.9%, 112/204) based on histological examination of tumor tissue by core needle biopsy or residual disease on surgical specimen.

### Pathological complete response

**Fig. 2:**
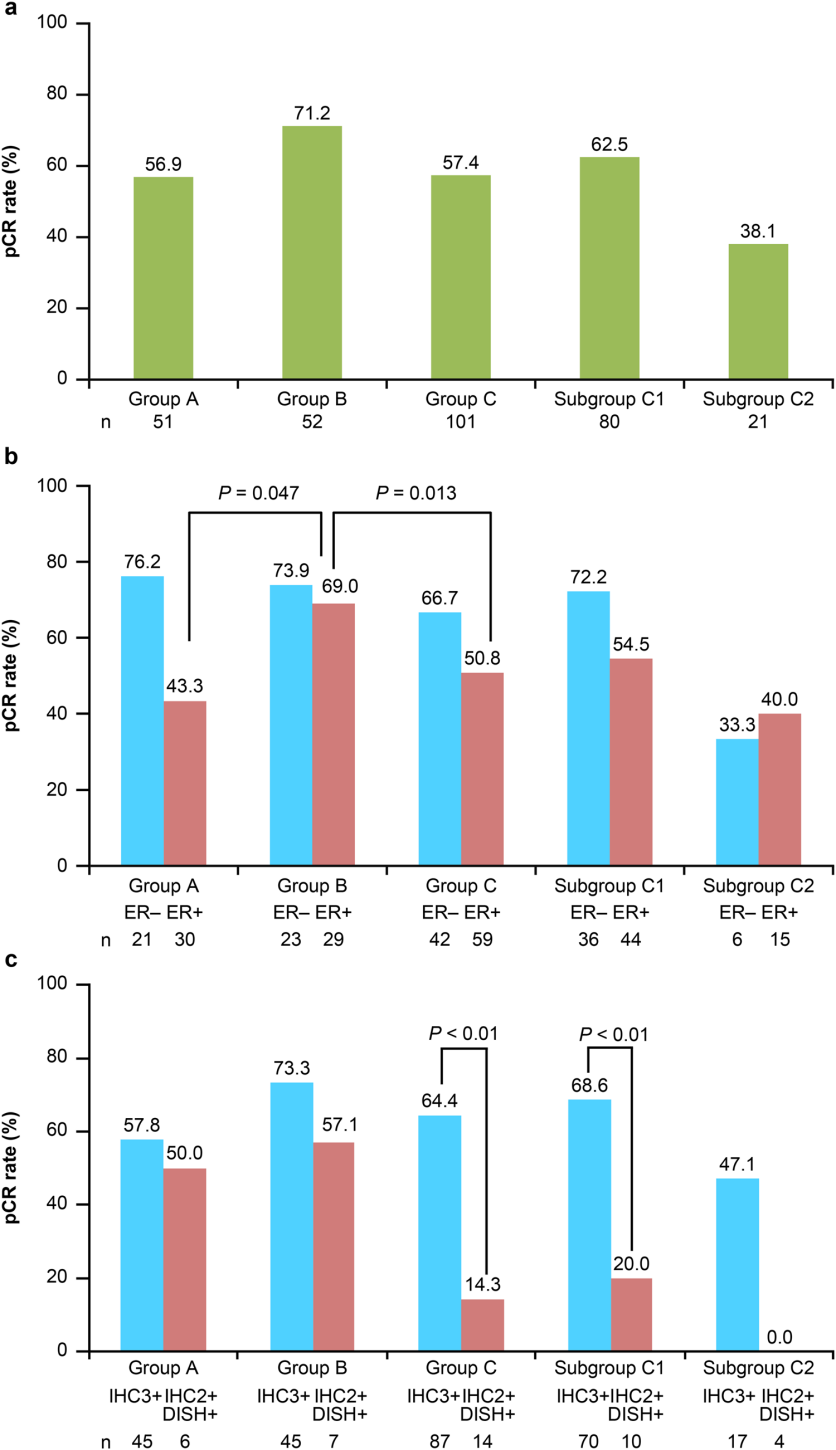
pCR rate in **a** all patient groups, **b** ER+/− patients, and **c** patients with strong/mild HER2 expression. *DISH* dual in situ hybridization, *ER* estrogen receptor, *HER2* human epidermal growth factor receptor 2, *IHC* immunohistochemistry, *IHC2+* equivocal for HER2 protein expression (circumferential membrane staining that is incomplete, weak, or moderate within > 10% of invasive tumor cells or complete and circumferential intense membrane staining within ≤10% of invasive tumor cells); *IHC3+* positive HER2 expression (circumferential membrane staining that is complete, intense, and in >10% of invasive tumor cells), *pCR* pathological complete response

pCR rate was numerically higher in group B (71.2%) than in groups A (56.9%) and C (57.4%); all between-group comparisons were not significant (*P* < 0.05, by chi-square test) except group B vs group C2 (*P* = 0.0086), and group C1 vs group C2 (*P* = 0.0441). The rates were comparable between groups A and C and subgroup C1. pCR rate was lowest (38.1%) in the T-DM1+P nonresponder subgroup C2 who were switched to FEC (Fig. 2a). Results of the exploratory analysis showed that the pCR rate was significantly higher in group B than in groups A (*P* = 0.047) and C (*P* = 0.013) in patients with ER+ but was comparable in patients with ER− (Fig. 2b). There were no significant differences between the groups for other secondary endpoints of pathological response rate (Table 2). Results of the exploratory analysis showed that pCR rates were significantly higher (*P* < 0.01) in patients with strong HER2 expression (HER2-IHC 3+) than those with mild HER2 expression (HER2-IHC 2+, chromogenic in situ hybridization [CISH]-positive) in group C (56/87 [64.4%] versus 2/14 [14.3%]) and subgroup C1 (48/70 [68.6%] versus 2/10 [20.0%]), respectively (Fig. 2c).

**Table 2:** Pathological response rate (full analysis set, *N* = 204), clinical response rate, and breast conservation rate

Variable	Group A (*n* = 51)	Group B *(n* = 52)	Group C (*n* = 101)	Subgroup C1 (*n* = 80)	Subgroup C2 *(n* = 21)
CpCR^a^	29 (56.9%)	37 (71.2%)	58 (57.4%)	50 (62.5%)	8 (38.1%)
95% CI	(42.2, 70.7)	(56.9, 82.9)	(47.2, 67.2)	(51.0, 73.1)	(18.1, 61.6)
SpCR^a^	22 (43.1%)	30 (57.7%)	43 (42.6%)	36 (45.0%)	7 (33.3%)
95% CI	(29.3, 57.8)	(43.2, 71.3)	(32.8, 52.8)	(33.8, 56.5)	(14.6, 57.0)
QpCR	39 (76.5%)	43 (82.7%)	68 (67.3%)	58 (72.5%)	10 (47.6%)
95% CI	(62.5, 87.2)	(69.7, 91.8)	(57.3, 76.3)	(61.4, 81.9)	(25.7, 70.2)
QpCR and ypN0	38 (74.5%)	40 (76.9%)	68 (67.3%)	58 (72.5%)	10 (47.6%)
95% CI	(60.4, 85.7)	(63.2, 87.5)	(57.3, 76.3)	(61.4, 81.9)	(25.7, 70.2)
ORR (by investigator’s assessment)	49 (96.1%)	45 (86.5%)	89 (88.1%)	71 (88.8%)	18 (85.7%)
95% CI	(86.5, 99.5)	(74.2, 94.4)	(80.2, 93.7)	(79.7, 94.7)	(63.7, 97.0)
cCR rate (by MRI/PET-CT)	24 (47.1%)	27 (51.9%)	39 (38.6%)	31 (38.8%)	8 (38.1%)
95% CI	(32.9, 61.5)	(37.6, 66.0)	(29.1, 48.8)	(28.1, 50.3)	(18.1, 61.6)
Breast conservation rate, *n*/*N*^b^ (%)	26/50 (52.0%)	27/52 (51.9%)	51/100 (51.0%)	43/79 (54.4%)	8/21 (38.1%)
Breast conservation rate in patients who had pre-planned mastectomy, *n*/*N*^b^ (%)	11/32 (34.4%)	12/31 (38.7%)	20/63 (31.7%)	18/49 (36.7%)	2/14 (14.3%)

*Bp* partial mastectomy, *Bq* quadrantectomy, *cCR* complete clinical response, *CI* confidence interval, *CpCR* comprehensive pCR, *pCR* pathological complete response, *MRI* magnetic resonance imaging, *ORR* overall response rate, *PET-CT* positron emission tomography-computed tomography, *QpCR* quasi pCR, *SpCR* strict pCR

^a^There were no patients with lymph node metastasis who achieved CpCR or SpCR. The rate of CpCR and CpCRypN0 was identical as was the rate of SpCR and SpCRypN0

^b^Patients who underwent Bp or Bq and had a negative margin were defined as successful breast conservation

### Clinical response

ORR was high and comparable (86–96%) among groups (Table 2), and disease progression was observed in 2 patients in group C (assessed by Response Evaluation Criteria In Solid Tumors [RECIST] v1.1). cCR rate was comparable between groups A (47%) and B (52%), but marginally lower in group C (39%) and did not differ in the response-guided subgroups C1 (39%) and C2 (38%) (Table 2). There was a decrease in tumor size from baseline at cycle 4 in all patients, except 1 each in group A and subgroup C1 (pCR achieved), and subgroup C2 (pCR not achieved) (Online Resource 4).

### Breast conservation rate

Breast conservation was achieved in approximately half (51–54%) of the patients in groups A, B, and C and subgroup C1; success rate was lower in subgroup C2 (38%). Similarly, among patients who underwent BCS instead of planned mastectomy, breast conservation success rate was higher in groups A, B, and C and subgroup C1 (32–39%) than subgroup C2 (14%) (Table 2).

### Disease-free survival and overall survival

At the median follow-up of 1064 days (range 705–1541 days), 3, 3, and 5 events of recurrence had been reported in groups A, B, and C, respectively, and 2, 2, and 3 patients in groups A, B, and C, respectively, had events of distant recurrence. Only 1 patient in group A had died due to breast cancer. The rate of DFS at 3 years was 94.3% in group A, 96.2% in group B, and 94.0% in group C. The rate of OS at 3 years was 99.2% among all patients.

### Safety

Treatment-related adverse events (AEs) by treatment group (≥ 10% incidence) are presented in Online Resource 5. Most commonly reported drug-related AEs (≥ 10% incidence and ≥ 10% difference between group A and subgroup C1) were alopecia (94.1% versus 5%), diarrhea (86.3% versus 32.5%), decrease in white blood cell count (86.3% versus 8.8%), and neutropenia (84.3% versus 20%) (Table 3). Most commonly reported drug-related AEs (≥ 10% incidence and ≥ 10% difference between group B and subgroup C1) were alopecia (86.5% versus 5%), neutropenia (76.9% versus 20%), and nausea (75% versus 50%). Grade 3/4 AEs were significantly less frequent in subgroup C1 (33.8%) than group A (84.3%), group B (76.9%), or subgroup C2 (76.2%; all *P* < 0.001). Grade 3/4 neutropenia, febrile neutropenia, and leukopenia were reported less often in subgroup C1 than groups A or B, or subgroup C2 (Online Resource 6). No unexpected drug-related AEs were reported. Drug-related alopecia and febrile neutropenia were less frequent in subgroup C1 (5.0%, 0%) than group A (94.1%, 21.6%), group B (86.5%, 15.4%), or subgroup C2 (81%, 33.3%). No treatment discontinuations or deaths due to AEs were reported.

**Table 3:** Drug-related adverse events (≥ 10% incidence and ≥ 10% difference between group A and subgroup C1 or group B and subgroup C1)

Drug-related adverse event	Overall, *n* (%) (*n* = 204)	Group A, *n* (%) (*n* = 51)	Group B, *n* (%) (*n* = 52)	Subgroup C1, *n* (%) (*n* = 80)	Subgroup C2, *n* (%) (*n* = 21)
Blood and lymphatic system disorders					
Neutropenia	107 (52.5)	43 (84.3)	40 (76.9)	16 (20.0)	8 (38.1)
Anemia	72 (35.3)	27 (52.9)	31 (59.6)	8 (10.0)	6 (28.6)
Febrile neutropenia	26 (12.7)	11 (21.6)	8 (15.4)	0 (0.0)	7 (33.3)
Metabolism and nutrition disorders					
Decreased appetite	74 (36.3)	25 (49.0)	21 (40.4)	19 (23.8)	9 (42.9)
Psychiatric disorders					
Insomnia	13 (6.4)	7 (13.7)	3 (5.8)	2 (2.5)	1 (4.8)
Nervous system disorders					
Dysgeusia	100 (49.0)	35 (68.6)	38 (73.1)	16 (20.0)	11 (52.4)
Neuropathy peripheral	64 (31.4)	25 (49.0)	28 (53.8)	8 (10.0)	3 (14.3)
Gastrointestinal disorders					
Nausea	136 (66.7)	39 (76.5)	39 (75.0)	40 (50.0)	18 (85.7)
Diarrhea	119 (58.3)	44 (86.3)	38 (73.1)	26 (32.5)	11 (52.4)
Stomatitis	111 (54.4)	40 (78.4)	31 (59.6)	25 (31.3)	15 (71.4)
Constipation	61 (29.9)	24 (47.1)	20 (38.5)	8 (10.0)	9 (42.9)
Vomiting	56 (27.5)	21 (41.2)	21 (40.4)	5 (6.3)	9 (42.9)
Abdominal pain upper	29 (14.2)	13 (25.5)	7 (13.5)	5 (6.3)	4 (19.0)
Skin and subcutaneous tissue disorders					
Alopecia	114 (55.9)	48 (94.1)	45 (86.5)	4 (5.0)	17 (81.0)
Dermatitis acneiform	44 (21.6)	17 (33.3)	13 (25.0)	11 (13.8)	3 (14.3)
Palmar-plantar erythrodysesthesia syndrome	28 (13.7)	13 (25.5)	14 (26.9)	0 (0.0)	1 (4.8)
Nail discoloration	22 (10.8)	9 (17.6)	13 (25.0)	0 (0.0)	0 (0.0)
Nail disorder	17 (8.3)	7 (13.7)	7 (13.5)	1 (1.3)	2 (9.5)
Musculoskeletal and connective tissue disorders					
Myalgia	31 (15.2)	9 (17.6)	16 (30.8)	4 (5.0)	2 (9.5)
Arthralgia	30 (14.7)	4 (7.8)	14 (26.9)	8 (10.0)	4 (19.0)
General disorders and administration site conditions					
Malaise	86 (42.2)	27 (52.9)	25 (48.1)	22 (27.5)	12 (57.1)
Pyrexia	41 (20.1)	15 (29.4)	12 (23.1)	5 (6.3)	9 (42.9)
Edema peripheral	31 (15.2)	17 (33.3)	12 (23.1)	0 (0.0)	2 (9.5)
Fatigue	24 (11.8)	8 (15.7)	7 (13.5)	4 (5.0)	5 (23.8)
Edema	11 (5.4)	7 (13.7)	4 (7.7)	0 (0.0)	0 (0.0)
Investigations					
Platelet count decreased	114 (55.9)	12 (23.5)	30 (57.7)	55 (68.8)	17 (81.0)
Alanine aminotransferase increased	98 (48.0)	20 (39.2)	23 (44.2)	42 (52.5)	13 (61.9)
White blood cell count decreased	97 (47.5)	44 (86.3)	38 (73.1)	7 (8.8)	8 (38.1)
Aspartate aminotransferase increased	91 (44.6)	15 (29.4)	22 (42.3)	41 (51.3)	13 (61.9)
Weight decreased	20 (9.8)	8 (15.7)	9 (17.3)	0 (0.0)	3 (14.3)
Blood alkaline phosphatase increased	19 (9.3)	1 (2.0)	4 (7.7)	13 (16.3)	1 (4.8)
Injury, poisoning, and procedural complications					
Infusion related reaction	89 (43.6)	10 (19.6)	21 (40.4)	45 (56.3)	13 (61.9)

## Discussion

Results of this study showed that the ypT0/is and ypN0 rate was numerically higher with the TCbHP →  T-DM1+P regimen (71%; group B) than with the standard TCbHP regimen (57%; group A) and the T-DM1+P regimen (57%; group C). As expected, in group C, the rate was higher among responders who continued T-DM1+P (63%; subgroup C1) than in nonresponders who switched to FEC (38%; subgroup C2). Notably, the pCR rate (57.4%) after the initial 4 cycles of T-DM1+P (group C) was equivalent to that with the standard TCbHP regimen (57%). Of note, the pCR rate in the ER+ subgroup of patients was significantly higher in group B (69%) than groups A (43.3%) and C (50.8%), but was comparable in ER– subgroups of patients (66.7–76.2%).

The overall rate of OS was 99.2% and rate of DFS was ≥ 94% in all groups at 3 years in our study, which are reflective of anthracycline-free post-operative therapy in 91.2% of patients in groups A and B. In group C, post-operative anthracyclines were used in approximately 60% of patients, suggesting that even if T-DM1-based pre-operative treatment is administered, anthracyclines can be avoided in 40% of the cases. However, further confirmation of long-term prognosis with anthracycline-free adjuvant therapy is needed.

We observed a remarkably high pCR rate in group B and hypothesize that the HER2+ cancer cells/clones that persisted [19] after the initial 4 cycles of TCbHP treatment may have responded to the subsequent 4 cycles of T-DM1+P. Interestingly, this sequence of TCbHP → T-DM1+P increased the pCR rate compared with the standard regimen of TCbHP, particularly for luminal HER2+ disease.

Although direct comparisons cannot be made with other studies due to differences in study design, region, and treatment regimens, the TRYPHAENA study reported a pCR rate (ypT0-isypN0) of 50.7% with the FEC+H+P×3 → T+H+P×3 regimen, 45.3% with the FEC×3 → T+H+P×3 regimen, and 51.9% with the TCH+P×6 regimen, the only comparable arm, compared with 57% in group A of this study [8]. The pCR rates in the ADAPT study were 41.0% for the T-DM1×4 regimen and 41.5% for the T-DM1+ET×4 regimen in patients with early HER2+ HR+ breast cancer [20]. However, in ADAPT, patients received 4 treatment cycles unlike 6 in our study. In the KRISTINE study, pCR was achieved in 44.4% (HR+, 38.1%; HR−, 54.8%) of patients in the T-DM1+P×6 group compared with 57.4% in group C in this study, and in 55.7% (46.4%, 71.1%) of patients in the TCbH+P×6 group, which was similar to group A of this study (57%) [10]. However, it should be noted that in our study, patients with ER+ breast cancer were treated with concurrent HT along with T-DM1+P, which could have additionally impacted the pCR rates. In an analysis of the I-SPY2 trial in invasive breast cancer in HER2+ subsets, neoadjuvant T-DM1+P×4 followed by doxorubicin + cyclophosphamide × 4 resulted in a pCR rate of 52% (HR+, 46%; HR−, 64%) versus 22% (HR+, 17%; HR−, 33%) with taxane (paclitaxel) + trastuzumab only. The results were similar to the response-guided group C and their ER status for this study [9]. With regard to the DFS and OS outcomes, less than 3 events of recurrence and no events of death were observed at the 3-year follow-up in group C treated with the T-DM1-based regimen. This good outcome is also supported by the results of the KRISTINE trial (T-DM1+P versus TCH + P) [21] and can be expected to spur the development of T-DM1-based pre-operative therapy. Similar to the current study, T-DM1-based regimens demonstrated favorable results in patients with HER2+ metastatic breast cancer in previous studies, including TDM4450g [22] and EMILIA [23]; the efficacy of T-DM1 was more evident in patients with high IHC (3+) [21] or HER2-mRNA [21, 23] expression.

The efficacy of T-DM1 is also supported by the KATHERINE study, where the interim analysis shows that the invasive DFS was significantly higher in the T-DM1 group than the trastuzumab alone group among patients with HER2+ early breast cancer with residual invasive disease after completion of neoadjuvant therapy [24].

In pre-menopausal women with ER+, ovarian suppression is often engaged using HT. In the ADAPT study, pCR rates were 41.5% and 15.1% in patients with HER2+ HR+ breast cancer receiving concomitant HT with 12-week T-DM1 and trastuzumab, respectively [20], suggesting improved efficacy of anti-HER2 therapy without any detrimental effects with concomitant HT. In the Neo-LaTH study [25], patients receiving anti-HER2 therapy (with/without HT) before chemotherapy for longer duration (18 versus 6 weeks) tended to show more tumor shrinkage. In our study as well, pCR was the highest (69%) in patients with ER+ in group B that received T-DM1 with concurrent HT, which was preceded by 4 cycles of TCbHP. Thus, de-escalation strategies using an antibody–drug conjugate regimen along with concomitant HT to enhance treatment efficacy warrant further investigation.

This approach of imaging-based and biopsy-incorporated treatment personalization may be useful for both maintaining pCR rates and minimizing toxicity. Further evaluation is required to support the initiation of the T-DM1+P with or without HT regimen, which may be useful in a selected group of HER2+ patients based on lack of feasibility.

As anticipated, patients with strong HER2 expression (IHC3+) had higher pCR rates than those with mild HER2 expression (IHC2+ and DISH+) in group C and subgroup C1, suggesting that the effect of T-DM1 might depend on membrane HER2 expression levels.

Breast conservation was achieved in approximately half (51–54%) of the patients, except in subgroup C2 (38%). Also, among those who underwent BCS instead of planned mastectomy, breast conservation success rate was higher in all patients (32–39%), except subgroup C2 (14%). These findings may be useful for considering surgical approaches for each individual. As anticipated, compared to the T-DM1 backbone only arm (group C), the incidence of AEs was highest in the taxane backbone arm (group A) followed by the taxane + T-DM1 backbone arm (group B). The T-DM1 backbone arm also reported better quality of life in the Swedish PREDIX HER2 trial (T-DM1 versus DTP) [26] and the KRISTINE trial (T-DM1+P versus TCH+P) [21]. Of special mention are drug-related alopecia and febrile neutropenia, which were less frequent in subgroup C1 (5.0%, 0%) versus group A (94.1%, 21.6%), group B (86.5%, 15.4%), or subgroup C2 (81%, 33.3%). This suggests that tailoring the T-DM1 backbone into the neoadjuvant therapy regimens is beneficial from a safety perspective and the treatment of HER2+ breast cancer can be modified with a more personalized approach. Study limitations include the fact that the pharmacoeconomic efficiency of the regimens and impact of the pCR rate of the T-DM1 regimen on long-term survival and quality of life were not evaluated.

## Conclusion

Overall, the pCR rate was not statistically different among the treatment groups. However, compared with the standard TCbHP and response-guided T-DM1+P regimens, the pCR rate with the TCbHP →  T-DM1+P regimen was numerically higher in the neoadjuvant setting. Exploratory analyses showed that the pCR rate was significantly higher with the TCbHP →  T-DM1+P regimen than with the standard TCbHP and T-DM1+P regimens in patients with ER+, but was comparable in ER− patients. ER status might be an indicator to personalize dual HER2-targeted therapy. T-DM1+P was generally safe and well tolerated, with the fewest AEs throughout the study. No significant differences in safety were observed between patients who received TCbHP alone and TCbHP → T-DM1+P. Given the patient numbers, the personalization of treatment showed that the efficacy among T-DM1+P regimen responders was better than that of the TCbHP regimen alone and could thus serve as a reasonable approach to minimize toxicity while maintaining efficacy.

## Electronic supplementary material

Below is the link to the electronic supplementary material.

Supplementary file1 (DOCX 2633 kb)

## Data Availability

Data archiving is not mandated but data will be made available on reasonable request.

## Abbreviations

AE: Adverse event
AI: Aromatase inhibitor
BCS: Breast-conserving surgery
Bp: Partial mastectomy
Bq: Quadrantectomy
cCR: Clinical complete response
CI: Confidence interval
CISH: Chromogenic in situ hybridization
CpCR: Comprehensive pCR
DISH: Dual in situ hybridization
DFS: Disease-free survival
ER: Estrogen receptor
FEC: 5-Fluorouracil + epirubicin + cyclophosphamide
FISH: Fluorescence in situ hybridization
HER2: Human epidermal growth factor receptor 2
HR: Hormone receptor
HT: Hormone therapy
IHC: Immunohistochemistry
JBCRG: Japan Breast Cancer Research Group
LHRH: Luteinizing hormone-releasing hormone
MedDRA/J: Medical dictionary for regulatory activities/japanese
MRI: Magnetic resonance imaging
NCI CTCAE: National cancer institute common terminology criteria for adverse events
ORR: Overall response rate
OS: Overall survival
pCR: Pathological complete response
pCRinv: Invasive pCR
PET-CT: Positron emission tomography-computed tomography
PFS: Progression-free survival
PgR: Progesterone receptor
QpCR: Quasi pCR
RDI: Relative dose intensity
RECIST: Response evaluation criteria in solid tumors
SpCR: Strict pCR
TCbHP: Docetaxel + carboplatin + trastuzumab + pertuzumab
T-DM1: Trastuzumab emtansine
T-DM1+P: T-DM1+Pertuzumab
