# Supplementary material for: A randomized, 3-arm, neoadjuvant, phase 2 study comparing docetaxel + carboplatin + trastuzumab + pertuzumab (TCbHP), TCbHP followed by trastuzumab emtansine and pertuzumab (T-DM1+P), and T-DM1+P in HER2-positive primary breast cancer
Source: Breast Cancer Res Treat. 2020 Jan 17;180(1):135–46. doi: 10.1007/s10549-020-05524-6 (PMC7031180; doi:10.1007/s10549-020-05524-6)
Supplement: Supplementary file 1 — Supplementary file1 (DOCX 2633 kb) [file 10549_2020_5524_MOESM1_ESM.docx]

A randomized, 3-arm, neoadjuvant, phase 2 study comparing docetaxel+carboplatin+trastuzumab+pertuzumab (TCbHP), TCbHP followed by trastuzumab emtansine and pertuzumab (T-DM1+P), and T-DM1+P in HER2-positive primary breast cancer

Norikazu Masuda^1^, Shoichiro Ohtani^2^, Toshimi Takano^3^, Kenichi Inoue^4^, Eiji Suzuki^5^, Rikiya Nakamura^6^, Hiroko Bando^7^, Yoshinori Ito^8^, Kazushige Ishida^9^, Takashi Yamanaka^10^, Katsumasa Kuroi^11^, Hiroyuki Yasojima^1^, Hiroi Kasai^12^, Tsuyoshi Takasuka^13^, Takaki Sakurai^14^, Tatsuki R. Kataoka^14^, Satoshi Morita^15^, Shinji Ohno^16^, Masakazu Toi^17^

^1^Department of Surgery, Breast Oncology, National Hospital Organization Osaka National Hospital, Osaka, Japan

^2^Department of Breast Surgery, Hiroshima City Hiroshima Citizens Hospital, Hiroshima, Japan

^3^Department of Medical Oncology, Toranomon Hospital, Tokyo, Japan

^4^Division of Breast Oncology, Saitama Cancer Center, Saitama, Japan

^5^Department of Breast Surgery, Kyoto University Hospital, Kyoto, Japan

^6^Division of Breast Surgery, Chiba Cancer Center, Chiba, Japan

^7^Breast and Endocrine Surgery, Faculty of Medicine, University of Tsukuba, Tsukuba, Japan

^8^Breast Medical Oncology Department, Cancer Institute Hospital of Japanese Foundation for Cancer Research, Tokyo, Japan

^9^Department of Surgery, Iwate Medical University, Morioka, Japan

^10^Department of Breast and Endocrine Surgery, Kanagawa Cancer Center, Yokohama, Japan

^11^Department of Breast Surgery, Tokyo Metropolitan Health and Hospitals Corporation Ebara Hospital, Tokyo, Japan

^12^Institute for Advancement of Clinical and Translational Science, Kyoto University Hospital, Kyoto, Japan

^13^Oncology Lifecycle Management Department, Chugai Pharmaceutical Co., Ltd., Tokyo, Japan

^14^Department of Diagnostic Pathology, Kyoto University Hospital, Kyoto, Japan

^15^Department of Biomedical Statistics and Bioinformatics, Kyoto University Graduate School of Medicine, Kyoto, Japan

^16^Breast Oncology Center, Cancer Institute Hospital of Japanese Foundation for Cancer Research, Tokyo, Japan

^17^Breast Cancer Unit, Graduate School of Medicine, Kyoto University Hospital, Kyoto University, Kyoto, Japan

**Corresponding author:**

Dr. Masakazu Toi

E-mail: [toi@kuhp.kyoto-u.ac.jp](mailto:toi@kuhp.kyoto-u.ac.jp)

**Online resource 1**. Study design

AUC, area under curve; cCR, clinical complete response; cPR, clinical partial response; CNB, core needle biopsy; ER, estrogen receptor; FEC, 5-fluorouracil+epirubicin+cyclophosphamide; HER2, human epidermal growth factor receptor 2; MRI, magnetic resonance imaging; R, randomization; TCbHP, docetaxel+carboplatin+trastuzumab+pertuzumab; T-DM1, trastuzumab emtansine; T‑DM1+P, trastuzumab emtansine+pertuzumab

**Online resource 2**

| **Treatment** |
| --- |
| Details of dose schedule and cycles of the 3 chemotherapy regimens   - Dosing was started within 14 days of secondary enrollment. - TCbHP regimen (for groups A and B)   - Patients received the regimen (pertuzumab, 840 mg/body for cycle 1, 420 mg/body for the following cycles; trastuzumab, 8 mg/kg for cycle 1, 6 mg/kg for the following cycles; docetaxel, 75 mg/m^2^; carboplatin, AUC6) intravenously for cycle 1 to cycle 6 (group A) or cycle 4 (group B) on day 1 of each cycle. Dose reduction for the duration of the infusion at the next dose was not allowed if an intolerable IRR was observed at or within 24 hours of dosing. - T-DM1+P regimen (for groups B and C)   - Patients received the regimen intravenously for cycles 5 to 8 (group B; T-DM1, 3.6 mg/kg; pertuzumab, 420 mg/body) or cycles 1 to 4 (group C; T-DM1, 3.6 mg/kg; pertuzumab, 840 mg/body for cycle 1, 420 mg/body for cycle 2, and thereafter) on day 1 of each cycle. In group C, after completion of cycle 4, responders continued the regimen until cycle 6. Dose reduction for the duration of the infusion at the next dose was not allowed if an IRR was observed at or within 24 hours of dosing. - FEC regimen (group C)   - After completion of cycle 4, nonresponders received the regimen (5-FU, 500 mg/m^2^; epirubicin, 100 mg/m^2^; cyclophosphamide, 500 mg/m^2^) intravenously for cycles 5 to 8. When AEs were observed, dosing was allowed to proceed to the next cycle with a dose reduction of epirubicin (75 or 60 mg/m^2^); treatment was discontinued if further reduction of epirubicin was required. - Concurrent hormone therapy   - ER+ patients received concurrent hormone therapy (pre-menopausal, intravenous leuprorelin [11.25 mg every 12 weeks] and oral tamoxifen [20 mg once daily]; post-menopausal, letrozole [2.5 mg once daily]) for cycles 5 to 8 (group B), cycles 1 to 6 (group C responders), or cycles 1 to 4 (group C nonresponders) until 1 week before surgery.   Discontinuation and treatment suspension criteria   - When a delay of ≥1 day in the dosing of any of the neoadjuvant drugs was required, dosing of all other drugs in that regimen was delayed accordingly. Initiation of the neoadjuvant therapy (excluding concurrent hormone therapy) was not allowed to be delayed for >2 cycles (9 weeks after the previous cycle). If a delay of >2 cycles was required, the drug causing the delay was discontinued. When intolerable AEs were observed and did not recover after symptomatic treatment or dose reduction, the patient discontinued the drug causing the delay but continued with other drugs. Investigators were allowed to reduce the dose or delay the cycle initiation of the neoadjuvant therapy to the predefined levels at their discretion. When trastuzumab or T-DM1 was discontinued, pertuzumab was also discontinued.   Total exposure, concomitant medications, and subsequent anticancer treatments   - Post-operative adjuvant therapy was performed by investigator choice according to the clinical guidelines. One-year treatment was recommended for trastuzumab (including neoadjuvant therapy or T-DM1 treatment period). When residual, invasive cancer was pathologically observed, addition of appropriate chemotherapy (e.g., anthracyclines) was recommended. In ER+ patients, hormone therapy was recommended for >5 years. Local radiation therapy was performed when deemed necessary. |
| **List of participating centers** |
| - Hiroshima City Hiroshima Citizens Hospital; Toranomon Hospital; Saitama Cancer Center; Kyoto University Hospital; University of Tsukuba Hospital; Chiba Cancer Center; National Hospital Organization Osaka National Hospital; The Cancer Institute Hospital Of JFCR; Kanagawa Cancer Center; Iwate Medical University; Gunma Prefectural Cancer Center; Tokyo Metropolitan Cancer and Infectious Diseases Center Komagome Hospital; National Hospital Organization Kyushu Cancer Center; National Hospital Organization Shikoku Cancer Center; Nihon University Itabashi Hospital; National Cancer Center Hospital; Aichi Cancer Center Hospital. |

AE, adverse event; 5-FU, 5-fluorouracil; ER, estrogen receptor; FEC, 5-fluorouracil+epirubicin+cyclophosphamide; IRR, infusion related reaction; TCbHP, docetaxel+carboplatin+trastuzumab+pertuzumab; T-DM1, trastuzumab emtansine; T‑DM1+P, trastuzumab emtansine+pertuzumab

Online resource 3. Patient disposition

FAS, full analysis set; FEC, 5-fluorouracil+epirubicin+cyclophosphamide; HER2, human epidermal growth factor receptor 2; TCbHP, docetaxel+carboplatin+trastuzumab+pertuzumab; T-DM1, trastuzumab emtansine; T-DM1+P, trastuzumab emtansine+pertuzumab

**Online resource 4**. Waterfall plots representing tumor shrinkage at cycle 4

pCR, pathological complete response

**Online resource 5**. Drug-related adverse events by treatment group (≥10% incidence; safety analysis set, *n*=204)

Drug-related adverse events with ≥10% incidence in any of the treatment group are listed.

| Drug-related adverse event | Overall, *n* (%)  (*n*=204) | Group A, *n* (%)  (*n*=51) | Group B, *n* (%)  (*n*=52) | Subgroup C1, *n*(%)  (*n*=80) | Subgroup C2, *n*(%)  (*n*=21) |
| --- | --- | --- | --- | --- | --- |
| Infections and infestations | | | | | |
| Paronychia | 19 (9.3) | 5 (9.8) | 3 (5.8) | 9 (11.3) | 2 (9.5) |
| Angular cheilitis | 7 (3.4) | 2 (3.9) | 1 (1.9) | 1 (1.3) | 3 (14.3) |
| Blood and lymphatic system disorders | | | | | |
| Neutropenia | 107 (52.5) | 43 (84.3) | 40 (76.9) | 16 (20.0) | 8 (38.1) |
| Anemia | 72 (35.3) | 27 (52.9) | 31 (59.6) | 8 (10.0) | 6 (28.6) |
| Febrile neutropenia | 26 (12.7) | 11 (21.6) | 8 (15.4) | 0 (0.0) | 7 (33.3) |
| Metabolism and nutrition disorders | | | | | |
| Decreased appetite | 74 (36.3) | 25 (49.0) | 21 (40.4) | 19 (23.8) | 9 (42.9) |
| Psychiatric disorders | | | | | |
| Insomnia | 13 (6.4) | 7 (13.7) | 3 (5.8) | 2 (2.5) | 1 (4.8) |
| Nervous system disorders | | | | | |
| Dysgeusia | 100 (49.0) | 35 (68.6) | 38 (73.1) | 16 (20.0) | 11 (52.4) |
| Neuropathy peripheral | 64 (31.4) | 25 (49.0) | 28 (53.8) | 8 (10.0) | 3 (14.3) |
| Headache | 35 (17.2) | 7 (13.7) | 7 (13.5) | 14 (17.5) | 7 (33.3) |
| Vascular disorders | | | | | |
| Vasculitis | 7 (3.4) | 1 (2.0) | 2 (3.8) | 1 (1.3) | 3 (14.3) |
| Respiratory, thoracic and mediastinal disorders | | | | | |
| Epistaxis | 64 (31.4) | 14 (27.5) | 15 (28.8) | 29 (36.3) | 6 (28.6) |
| Gastrointestinal disorders | | | | | |
| Nausea | 136 (66.7) | 39 (76.5) | 39 (75.0) | 40 (50.0) | 18 (85.7) |
| Diarrhea | 119 (58.3) | 44 (86.3) | 38 (73.1) | 26 (32.5) | 11 (52.4) |
| Stomatitis | 111 (54.4) | 40 (78.4) | 31 (59.6) | 25 (31.3) | 15 (71.4) |
| Constipation | 61 (29.9) | 24 (47.1) | 20 (38.5) | 8 (10.0) | 9 (42.9) |
| Vomiting | 56 (27.5) | 21 (41.2) | 21 (40.4) | 5 (6.3) | 9 (42.9) |
| Abdominal pain upper | 29 (14.2) | 13 (25.5) | 7 (13.5) | 5 (6.3) | 4 (19.0) |
| Hepatobiliary disorders | | | | | |
| Hepatic function abnormal | 19 (9.3) | 2 (3.9) | 5 (9.6) | 11 (13.8) | 1 (4.8) |
| Skin and subcutaneous tissue disorders | | | | | |
| Alopecia | 114 (55.9) | 48 (94.1) | 45 (86.5) | 4 (5.0) | 17 (81.0) |
| Dermatitis acneiform | 44 (21.6) | 17 (33.3) | 13 (25.0) | 11 (13.8) | 3 (14.3) |
| Rash maculo-papular | 35 (17.2) | 8 (15.7) | 13 (25.0) | 13 (16.3) | 1 (4.8) |
| Rash | 30 (14.7) | 7 (13.7) | 10 (19.2) | 10 (12.5) | 3 (14.3) |
| Palmar-plantar erythrodysesthesia syndrome | 28 (13.7) | 13 (25.5) | 14 (26.9) | 0 (0.0) | 1 (4.8) |
| Nail discoloration | 22 (10.8) | 9 (17.6) | 13 (25.0) | 0 (0.0) | 0 (0.0) |
| Nail disorder | 17 (8.3) | 7 (13.7) | 7 (13.5) | 1 (1.3) | 2 (9.5) |
| Musculoskeletal and connective tissue disorders | | | | | |
| Myalgia | 31 (15.2) | 9 (17.6) | 16 (30.8) | 4 (5.0) | 2 (9.5) |
| Arthralgia | 30 (14.7) | 4 (7.8) | 14 (26.9) | 8 (10.0) | 4 (19.0) |
| General disorders and administration site conditions | | | | | |
| Malaise | 86 (42.2) | 27 (52.9) | 25 (48.1) | 22 (27.5) | 12 (57.1) |
| Pyrexia | 41 (20.1) | 15 (29.4) | 12 (23.1) | 5 (6.3) | 9 (42.9) |
| Edema peripheral | 31 (15.2) | 17 (33.3) | 12 (23.1) | 0 (0.0) | 2 (9.5) |
| Fatigue | 24 (11.8) | 8 (15.7) | 7 (13.5) | 4 (5.0) | 5 (23.8) |
| Edema | 11 (5.4) | 7 (13.7) | 4 (7.7) | 0 (0.0) | 0 (0.0) |
| Investigations | | | | | |
| Platelet count decreased | 114 (55.9) | 12 (23.5) | 30 (57.7) | 55 (68.8) | 17 (81.0) |
| Alanine aminotransferase increased | 98 (48.0) | 20 (39.2) | 23 (44.2) | 42 (52.5) | 13 (61.9) |
| White blood cell count decreased | 97 (47.5) | 44 (86.3) | 38 (73.1) | 7 (8.8) | 8 (38.1) |
| Aspartate aminotransferase increased | 91 (44.6) | 15 (29.4) | 22 (42.3) | 41 (51.3) | 13 (61.9) |
| Gamma-glutamyltransferase increased | 21 (10.3) | 3 (5.9) | 3 (5.8) | 10 (12.5) | 5 (23.8) |
| Weight decreased | 20 (9.8) | 8 (15.7) | 9 (17.3) | 0 (0.0) | 3 (14.3) |
| Blood alkaline phosphatase increased | 19 (9.3) | 1 (2.0) | 4 (7.7) | 13 (16.3) | 1 (4.8) |
| Injury, poisoning and procedural complications | | | | | |
| Infusion related reaction | 89 (43.6) | 10 (19.6) | 21 (40.4) | 45 (56.3) | 13 (61.9) |

**Online resource 6**. Grade 3/4 adverse events by treatment group (≥5% incidence; safety analysis set, *n*=204)

| Adverse event | Overall, *n* (%)  (*n*=204) | Group A, *n* (%)  (*n*=51) | Group B, *n* (%)  (*n*=52) | Subgroup C1, *n* (%)  (*n*=80) | Subgroup C2, *n* (%)  (*n*=21) |
| --- | --- | --- | --- | --- | --- |
| Blood and lymphatic system disorders | | | | | |
| Neutropenia | 83 (40.7) | 42 (82.4) | 36 (69.2) | 1 (1.3) | 4 (19.0) |
| Anemia | 8 (3.9) | 4 (7.8) | 2 (3.8) | 2 (2.5) | 0 (0.0) |
| Febrile neutropenia | 26 (12.7) | 11 (21.6) | 8 (15.4) | 0 (0.0) | 7 (33.3) |
| Metabolism and nutrition disorders | | | | | |
| Decreased appetite | 7 (3.4) | 4 (7.8) | 3 (5.8) | 0 (0.0) | 0 (0.0) |
| Vascular disorders |  |  |  |  |  |
| Gastrointestinal disorders | | | | | |
| Nausea | 6 (2.9) | 4 (7.8) | 1 (1.9) | 0 (0.0) | 1 (4.8) |
| Diarrhea | 7 (3.4) | 3 (5.9) | 4 (7.7) | 0 (0.0) | 0 (0.0) |
| General disorders and administration site conditions | | | | | |
| Fatigue | 5 (2.5) | 3 (5.9) | 1 (1.9) | 0 (0.0) | 1 (4.8) |
| Investigations | | | | | |
| Platelet count decreased | 34 (16.7) | 0 (0.0) | 10 (19.2) | 17 (21.3) | 7 (33.3) |
| Alanine aminotransferase increased | 16 (7.8) | 2 (3.9) | 6 (11.5) | 7 (8.8) | 1 (4.8) |
| White blood cell count decreased | 66 (32.4) | 35 (68.6) | 28 (53.8) | 0 (0.0) | 3 (14.3) |
| Gamma-glutamyltransferase increased | 6 (2.9) | 0 (0.0) | 1 (1.9) | 3 (3.8) | 2 (9.5) |
